# Negative Parenting, Adolescents’ Emotion Regulation, Self-Efficacy in Emotion Regulation, and Psychological Adjustment

**DOI:** 10.3390/ijerph19042251

**Published:** 2022-02-16

**Authors:** Laura Di Giunta, Carolina Lunetti, Giulia Gliozzo, W. Andrew Rothenberg, Jennifer E. Lansford, Nancy Eisenberg, Concetta Pastorelli, Emanuele Basili, Irene Fiasconaro, Eriona Thartori, Ainzara Favini, Alessia Teresa Virzì

**Affiliations:** 1 Department of Psychology, Sapienza University of Rome, 00185 Rome, Italy; carolina.lunetti@uniroma1.it (C.L.); giulia.gliozzo@uniroma1.it (G.G.); concetta.pastorelli@uniroma1.it (C.P.); emanuele.basili@uniroma1.it (E.B.); irene.fiasconaro@uniroma1.it (I.F.); eriona.thartori@uniroma1.it (E.T.); ainzara.favini@uniroma1.it (A.F.); virzi.1889458@studenti.uniroma1.it (A.T.V.); 2 Center for Child and Family Policy, Duke University, Durham, NC 27708, USA; rothenbergdrew@gmail.com (W.A.R.); lansford@duke.edu (J.E.L.); 3 Department of Psychology, Arizona State University, Tempe, AZ 85281, USA; nancy.eisenberg@asu.edu

**Keywords:** self-efficacy, emotion regulation, adolescence, parenting, maladjustment

## Abstract

This study examines associations between parents’ rejection and control, adolescents’ self-efficacy in their regulation of negative emotions, and maladjustment. Path analyses were employed to test (a) whether adolescents’ dysregulation and self-efficacy regarding anger/sadness regulation mediate the relationship between parental rejection/control and adolescent maladjustment; (b) whether adolescent adjustment mediates the association between parental rejection/control and dysregulation and self-efficacy regarding anger/sadness regulation. Participants included 103 Italian adolescents (Time 1: M age = 15.57; 53% male), their mothers (*n* = 103), and their fathers (*n* = 79). Follow up data were assessed one year later (Time 2). At Time 1, adolescent reports of the frequency of mothers’ and fathers’ rejection and control were examined. At Time 2, adolescent-reports of their beliefs about self-efficacy in regulating anger and sadness, as well as anger and sadness dysregulation, were assessed by two methods: questionnaire and mobile ecological momentary assessment. At Time 2, mothers’, fathers’, and adolescents’ reports of adolescents’ aggressive behaviors and depressive problems were also assessed. Maternal rejection was associated with higher one year later aggressive problems, which in turn were associated with higher dysregulation of sadness, and lower self-efficacy in dealing with both anger and sadness. In addition, maternal rejection was associated with higher depressive symptoms one year later, which in turn were associated with lower self-efficacy in dealing with sadness and higher dysregulation of both anger and sadness. Finally, maternal control was associated with higher depressive symptoms, whereas paternal control was associated with lower depressive symptoms.

## 1. Introduction

The transitional stage to adolescence is characterized by a significant increase in the prevalence of externalizing and internalizing problems that are associated with several long-term implications for psychosocial adjustment [1]. In this regard, emotion regulation appears to be an especially relevant aspect of self-regulation [2]. Because it is unlikely that people can efficiently regulate their emotions if they do not believe themselves capable of managing their emotions, it is important to consider beliefs about self-efficacy in emotion regulation. How do adolescents develop their self-efficacy beliefs in dealing with negative emotions? One answer to this question may come from understanding the impact that their parents’ behaviors have on such beliefs. The present study investigates the joint contribution to adolescent adjustment of parenting, emotion regulation and self-efficacy in emotion regulation.

### 1.1. Anger and Sadness Regulation and Adolescents’ Adjustment

Emotion regulation is a process which includes initiating, inhibiting, avoiding, maintaining, or modulating feelings, cognitions, and behaviors in the service of social competency [3]. Patterns of emotion dysregulation have been related to both externalizing [4,5] and internalizing behaviors [6,7]. Emotion regulation has received considerable attention in both prevention and intervention research, considering its fundamental role in the onset of psychopathological problems [8,9]. However, the association between emotion regulation and (mal)adjustment may depend on the type of emotion [10]. Negative emotions, such as anger and sadness, have received the greater part of this empirical attention, especially in children and adolescents. In particular, during childhood and adolescence, frequent anger and poor anger regulation, as well as higher sadness experience and lower sadness regulation, have been associated with aggressive and depressive symptoms, respectively [11,12,13]. However, maladaptive management of anger has also been associated with internalizing problems, and sadness dysregulation has also been linked to externalizing behaviors [14,15].

Research on adolescents’ daily emotional experiences and daily emotion regulation highlights the importance of studying the day-to-day variability of youths’ emotional experiences [16,17,18,19]. Advantages of the ecological momentary assessment (EMA) approach over traditional approaches in assessing emotional experience include higher ecological validity and more reliable measures than traditional measures; minimization of retrospective reporting biases; greater generalizability of results; and information about the social contexts associated with emotional experiences. However, some limitations have been identified in the aforementioned studies, such as the importance of moving from a focus on global negative emotionality to discrete emotions [17].

### 1.2. Self-Efficacy Regarding Anger/Sadness Regulation and Adolescents’ Adjustment

Empirical evidence supports cross-cultural associations between self-efficacy in dealing with negative emotions and both internalizing and externalizing problems during adolescence [20,21,22,23]. Caprara et al. (2008) [24] found in a young adult sample that self-efficacy regarding sadness regulation was more highly negatively associated with shyness and anxiety/depression and positively associated with self-esteem and positive affect than self-efficacy in dealing with anger. In comparison to self-efficacy regarding sadness, self-efficacy in anger regulation was more highly negatively associated with irritability and aggression. Moreover, among pre-adolescents from Italy, United States, and Colombia et al. (2017; 2018) [25,26] found that higher self-efficacy in regulating anger was related to lower externalizing problems, whereas greater self-efficacy beliefs regarding sadness regulation were associated with fewer internalizing problems. Longitudinal bidirectional associations have been found between pre-adolescent self-efficacy regarding anger regulation and both prior and subsequent youth emotional and behavioral problems [26]. It is possible that youths with higher internalizing and externalizing problems have lower mastery experiences regarding their abilities to deal with sadness and anger, which in turn may affect whether they believe themselves to be capable of handling their feelings in difficult situations.

### 1.3. Maternal and Paternal Rejection and Control and Adolescents’ Adjustment

Parental rejection includes high levels of hostility, undifferentiated rejection, and neglect and low levels of warmth in the relationship with their children [27,28,29]. Children and adolescents who have parents with high levels of rejection (i.e., low in warmth) have worse psychosocial outcomes than those who perceive their parents as being low in rejection [30]. A different and less coherent scenario exists for parental control. There is a robust link between psychological control (i.e., parents’ attempts to control adolescents’ thoughts and feelings) and maladjustment [31,32]. In contrast, the association is more variable between behavioral control (i.e., parents’ attempts to know and potentially to redirect their adolescents’ behavior) and youth adjustment [33]. Although mothers’ warmth and control are often positively associated with fathers’ warmth and control [34], fathers make a unique and independent contribution to their children’s social development, even after accounting for mothers’ contribution [35]. However, few studies have examined separate contributions of multiple aspects of mothers’ and fathers’ parenting (e.g., rejection, warmth, control) on adolescent adjustment. The present study analyzes the relations between parents’ rejection and control and adolescent aggressive problems and depressive symptoms in a country that is less represented in the scientific literature, specifically Italy. 

### 1.4. Parental Rejection and Control, Emotion Regulation, and Adolescents’ Adjustment

Previous research suggests that parenting contributes to adolescents’ emotion regulation, which in turn affects adolescent adjustment. For example, parental comforting of children when they show negative emotions is associated with fewer child difficulties in anger regulation [36]. Moreover, high positive parental affect (i.e., high warmth, low rejection) and low levels of parental control are associated with children’s adaptive emotional regulation [37]. Negative controlling parenting styles are associated with fewer capabilities for children to shift their attention to less emotionally distressing events and with higher levels of child negative affect, which in turn are associated with higher problematic behaviors [38].

### 1.5. The Present Study 

This study investigates whether adolescents’ regulation of specific negative emotions and self-efficacy beliefs in dealing with such emotions mediate the relation between parental rejection and control and adolescents’ aggressive behaviors and depressive symptoms. Two negative emotions have been examined separately: anger and sadness. This study has three principal hypotheses: (a) adolescents’ anger and sadness dysregulation and adolescents’ low self-efficacy beliefs about their ability to deal with anger and sadness will be associated with more aggressive behaviors and depressive symptoms. (b) High maternal and paternal rejection and high maternal and paternal control will be related to adolescents’ anger and sadness dysregulation and to adolescents’ low self-efficacy in regulating anger and sadness. (c) Adolescents’ anger and sadness regulation and correspondent self-efficacy beliefs will mediate the relation between maternal and paternal rejection and control and adolescents’ aggressive behaviors and depressive symptoms. Because the emotion regulation constructs, aggressive behavior, and depressive symptoms were measured concurrently, we also tested an alternative hypothesis, that adolescents’ aggressive behaviors and depressive symptoms will mediate the relation between maternal and paternal rejection and control and adolescents’ anger and sadness regulation and correspondent self-efficacy beliefs. 

## 2. Materials and Methods

### 2.1. Participants and Procedure

Participants were part of the longitudinal Parenting Across Cultures Study (PAC) [39]. At Time 1, participants were 103 adolescents (*M_age_* = 15.56, *SD* = 0.77; 53% male), their mothers (*n* = 103), and their fathers (*n* = 79) from Rome, Italy. At Time 2, participants included 102 adolescents (*M_age_* = 16.77, *SD* = 0.78), their mothers (*n* = 100), and their fathers (*n* = 76). Mothers averaged 13.88 years of education (*SD* = 4.25), and fathers averaged 13.46 years of education (*SD* = 4.24). In line with MacCallum, Browne, and Sugawara (1996), a power of 61% is achieved with SEM implemented with *N*=100, whereas a power of 80% is achieved with *N* = 132. Families were recruited until the target sample size of around 100 families was reached, from schools serving high-, middle-, and low-income families in the approximate proportion to which these groups matched the socio-demographic characteristics of Italian society.

The data collection considered in this study refers to PAC’s Time 8. At that time, all participants were very much familiar with the overall annual research procedure and the team of researchers. Once per year, researchers contacted each family member, informed them about the characteristics of the research procedure, and were always open to answer any participants’ question about the research project and, in case of a request of support for any family member’s well-being, to refer them to the Counselling and Clinical Services at Sapienza University of Rome.

Once having obtained parental informed consent and child assent, interviews were conducted in the participant’s home or a location of their choosing. Interviews lasted approximately one hour. Parents were given modest financial compensation for their participation, and youths were given a small age-appropriate gift. When adolescents were 16 (i.e., PAC’s Time 8), the mobile ecological momentary assessment (mEMA) was employed [40]. Specifically, for 15 consecutive days, the youths received three e-mails (in the morning, afternoon, and evening) via their own mobile phones. Each e-mail was personalized for participants and contained a URL link that comprised questions concerning the participants’ current mood and thinking. On average, each data completion took 5 min. Taken together, the adolescents received 4635 emails. All of the questions included in the mEMA protocol were completed 4086 times, which corresponds to a completion rate of 88%. 

### 2.2. Measures 

#### 2.2.1. Parents’ Rejection and Control

The Parental Acceptance–Rejection/Control Questionnaire-Short Form (PARQ/Control-SF) [41] was used to assess youth reports of their mothers’ and fathers’ behaviors. Youths rated 17 items (1 = never or almost never, 4 = every day) [27,28]. We used the total acceptance–rejection scale by averaging 6 warmth–affection (e.g., “My mother/father says nice things to me,” reverse scored), 4 hostility–aggression (e.g., “My mother/father punishes me severely when (s)he is angry”), 4 rejection (e.g., “My mother/father seems to dislike me”), and 3 neglect–indifference items (e.g., “My mother/father pays no attention to me”). In addition, youths rated 3 items that were averaged to refer to a composite score for control (e.g., “My mother/father wants to control whatever I do”). Alphas were 0.87 and 0.90 for the total rejection score and 0.61 and 0.68 for the control score referring to mothers and fathers, respectively.

#### 2.2.2. Anger and Sadness Dysregulation

We employed two methods to assess anger and sadness dysregulation: questionnaires and mEMA. Youths rated (1 = almost always untrue of you to 5 = almost always true of you) subscales from the Early Adolescent Temperament Questionnaire-Revised (EATQ-R) [42] about their irritability (6 items, e.g., “I get irritated when I have to stop doing something that I am enjoying,” α = 0.73) and their sadness (4 items, e.g., “I feel depressed when unable to accomplish some task,” α = 0.86). 

In the mEMA procedure, youths were asked 3 times a day (morning, afternoon, and evening) for 15 days whether they were feeling angry, mad, and irritated in that moment (3 items; 1 = very slightly or not at all to 5 = extremely). Alpha among those three items was 0.97; thus, they were averaged to create a composite score for anger dysregulation via mEMA. A similar procedure was employed to create an mEMA sadness dysregulation score with the three items related to feeling sad, discouraged, and dejected in the moment (3 items; α = 0.96).

#### 2.2.3. Adolescents’ Self-Efficacy Beliefs Regarding Anger and Sadness Regulation

We also employed two methods to assess self-efficacy in dealing with anger and sadness: questionnaires and mEMA. Youths rated (1 = not well at all to 5 = very well) their ability to deal with anger and sadness with the Regulatory Emotional Self-Efficacy Scale (self-efficacy about anger regulation: 3 items, e.g., “How well can you keep from getting angry when others unfairly treat you badly?”; α = 0.83; self-efficacy regarding sadness regulation: 3 items, e.g., “How well can you avoid getting discouraged if your friends or significant others are not there when you need them?”; α = 0.83) [24,25]. In the mEMA procedure, if youths reported a score above 1 in at least one of the three anger-related items indicating that they were feeling anger in that moment, they were directed to answer the following question: “How well do you think you are capable of dealing with your anger or irritability in this moment?” (1 item; 1 = not well at all to 5 = very well). A similar procedure was employed to assess mEMA self-efficacy regarding sadness regulation (1 item; “How well do you think you are capable of dealing with your sadness or discouragement in this moment?”; 1 = not well at all to 5 = very well).

#### 2.2.4. Adolescents’ Aggressive Behaviors and Depressive Symptoms

At age 16 parents and youths, respectively, completed the Child Behavior Checklist (CBCL) and Youth Self-Report (YSR) [43]. We focused on two composite scores: aggressive behaviors (20 items in CBCL and 19 items in YSR; e.g., “My child gets in many fights” or “I get in many fights”) and depressive symptoms (5 items in both CBCL and YSR; e.g., “My child cries a lot” or “I cry a lot”). Parents and youths indicated whether each of the target behavior was not true (coded as 0), somewhat or sometimes true (coded as 1), or very true or often true (coded as 2). 

For youths’ aggressive behaviors, alpha was 0.79, 0.84, and 0.81 for youths’, mothers’, and fathers’ reports, respectively. For youths’ depressive symptoms, alpha was 0.76, 0.69, and 0.76 for youths’, mothers’, and fathers’ reports, respectively. For youths’ aggressive behaviors, the correlations between mothers’ and youths’ reports, fathers’ and youths’ reports, and between mothers’ and fathers’ reports were 0.28, 0.40, and 0.62, respectively. Correspondent correlations for youths’ depressive symptoms were 0.51, 0.39, and 0.47, respectively. Despite previous studies suggesting low inter-informant agreement in youth internalizing problems and moderate-to-strong agreement in youth externalizing problems [44], in this study a moderate-to-strong inter-informant agreement emerged in both adolescents’ aggressive behaviors and depressive symptoms. Thus, in line with empirical evidence underlying the significant advantages of multiple-informant designs [45], we created multi-informant composite scores for those outcomes.

#### 2.2.5. Covariates

Maternal and paternal education, and youths’ age, gender, and social desirability were included as covariates. In particular, social desirability referred to a subscale (10 items; 1 = very false for me, 5 = very true for me; e.g., “I’ve always gotten along with everyone”; α = 0.69) of the Big Five Questionnaire [46].

### 2.3. Analytic Plan

Preliminary descriptive statistics and Pearson’s correlations were examined. A measurement model to examine the possibility of creating a unique factor for anger dysregulation based on the two methods of questionnaires and mEMA was examined via confirmatory factor analysis (CFA). Similar multi-method CFA models were also examined for sadness dysregulation, and self-efficacy in dealing with anger and sadness, respectively. 

Then, we examined two series of developmental path models. The first series of models focused on the association between mothers’ and fathers’ rejection and control (youth aged 15), youths’ regulation and self-efficacy regarding anger (youth aged 16), and youth adjustment (aggressive behaviors and depressive symptoms at age 16). Because anger-related measures and the outcomes were assessed concurrently, we examined both the paths from parenting measures to youth anger-regulation-related measures, which in turn were associated with youth adjustment, as well as the paths from parenting measures to youth (mal)adjustment, which in turn were associated with youth anger-regulation-related measures. The Akaike Information Criterion (AIC; the lower the AIC index, the better the goodness-of-fit) [47] was used to compare the fit of these competing models. The second series of models mirrored the aforementioned series, but this time, instead of anger-related measures, the focus was on sadness-related measures. All models controlled for maternal and paternal education, youths’ age, gender, and social desirability.

Full information maximum likelihood (FIML) [48] within Mplus 7 (Muthen & Muthen Company, Los Angeles, CA, USA) [49] was used to account for missing data. We allowed all measures to covary within waves. A model was considered to have good fit if the χ^2^ test was nonsignificant (*p* ≥ 0.05), the CFI ≥ 0.95, the RMSEA ≤ 0.06, and the SRMR ≤ 0.08 [47]. Lastly, we followed the asymmetric confidence interval method recommended by MacKinnon et al. (2002) to formally test the mediated effects.

## 3. Results

### 3.1. Descriptive Statistics and Correlations

In the two last bottom lines of Table 1, descriptive statistics for the overall sample, and separately for mothers, fathers, and youths are reported. Univariate normality of variables was examined, and none of the variables was found to have univariate skewness > 2.0 and kurtosis > 7.0. Correlations among the examined variables for the overall sample are also reported in Table 1. 

Considering within-parenting style correlations, positive correlations emerged between maternal and paternal rejection, and between maternal and paternal control. Negative correlation emerged between maternal rejection and paternal control. Considering within-emotion-regulation-related correlations, different correlational patterns were identified. Within the same method (i.e., relying on scores based on questionnaires and mEMA, separately), dysregulation scores were all negatively and moderately associated with correspondent self-efficacy scores (e.g., r = −0.21 for the correlation between anger dysregulation and self-efficacy beliefs regarding anger regulation). Within the same emotion, positive and weak to moderate correlations emerged across methods within the same emotion (ranging from r = 0.16 for the correlation between youths’ anger dysregulation assessed via questionnaire and via mEMA to r = 0.33 for the correlation between youths’ self-efficacy regarding anger regulation assessed via questionnaire and via mEMA). Moreover, moderate to strong correlations emerged across emotions within the same method (e.g., r = 0.39 for the correlation between youths’ anger and sadness, both assessed via questionnaire; and r = 0.89 for the correlation between youths’ anger and sadness dysregulation, both assessed via mEMA). Considering within-youth adjustment correlations, aggressive behaviors were strongly and positively related to each other. Considering the correlations between parenting styles and youth adjustment, positive and moderate correlations emerged between maternal and paternal rejection and youth adjustment. Considering the correlations between parenting styles and emotion-regulation-related indicators, only paternal control was positively related to youths’ anger dysregulation (assessed both with questionnaires and mEMA). Maternal and paternal rejection were positively associated with sadness dysregulation, although assessed only with questionnaires. In addition, maternal rejection emerged as the only factor correlated with self-efficacy beliefs in dealing with sadness assessed with both questionnaire and mEMA. Finally, considering the correlations between emotion-regulation-related indicators and youth adjustment, whereas depressive symptoms were moderately related to anger and sadness dysregulation via both methods, aggressive behaviors were moderately related only with anger and sadness dysregulation via questionnaire. Moreover, aggressive behaviors were moderately and negatively related with self-efficacy beliefs in dealing only with anger via both methods, whereas depressive symptoms were moderately and negatively related with self-efficacy beliefs in dealing with both anger and sadness, but only via questionnaire. 

Among the aforementioned correlations, there was one correlation that was very strong (r = 0.89 for the correlation between anger and sadness dysregulation via mEMA). We reasoned that this correlation was mainly due to shared variance due to the same method (i.e., it examined the correlation between the same items asked for three times a day, for 15 days). Thus, for the sake of clarity in the interpretation of the results, the following analyses examined the association between regulation and self-efficacy beliefs maintaining separate models for anger and sadness. 

### 3.2. Measurement Model for Anger and Sadness Dysregulation and for Self-Efficacy Beliefs in Dealing with Anger and Sadness

Two CFA models were examined to test the association between regulation and self-efficacy beliefs within each emotion. The first CFA model concerned anger-related measures; the second concerned sadness-related measures. In both models, a two oblique factor model was examined in which each latent factor (e.g., multi method anger dysregulation and multi-method self-efficacy regarding anger regulation) had two indicators (namely, the two methods with which each correspondent construct was assessed: via questionnaire and via mEMA). 

**Figure 1:**
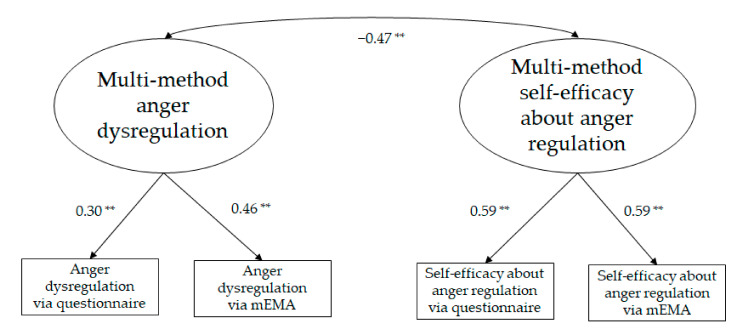
Measurement model for the anger-related measures (** = *p* ≤ 0.01).

The model for anger-related measures (Figure 1) fitted the data, χ^2^(2) = 3.27, *p* = 0.20, CFI = 0.93, RMSEA = 0.07, 90% CI = 0.00; 0.23, *p* = 0.27; SRMR = 0.05. Modification indices suggested inclusion in the model of a path between the two indicators that were assessed via mEMA. However, this path ended up being not significant and, thus, it was not included in Figure 1.

**Figure 2:**
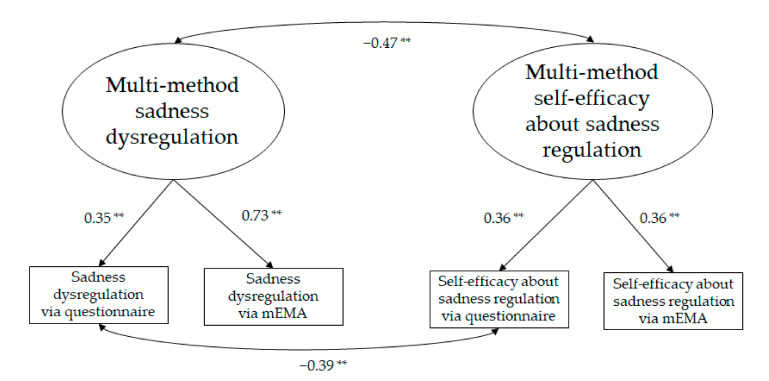
Measurement model for sadness-related measures (** *p* ≤ 0.01).

The model concerning sadness-related measures (Figure 2) also fitted the data, χ^2^(2) = 3.96, *p* = 0.14, CFI = 0.95, RMSEA = 0.09, 90% CI = 0.00; 0.24, *p* = 0.21; SRMR = 0.06. Modification indices suggested inclusion of a path between the two indicators that were assessed via questionnaire. This path was significant and negative, indicating that there was still some factor that those two indicators had in common, beyond the variance each of them shared with its own latent factor.

On the basis of these CFAs, we standardized all of the measures related to regulation and self-efficacy regarding anger and sadness, and we averaged each score within the same emotion and across the multiple methods. In Table 2, the correlations between the examined variables including the brand-new multi-method scores for regulation and self-efficacy beliefs concerning anger and sadness are reported. The following analyses were performed considering the multi-method scores.

### 3.3. Maternal and Paternal Rejection and Control → Regulation and Self-Efficacy Regarding Anger → Youth Adjustment

**Figure 3:**
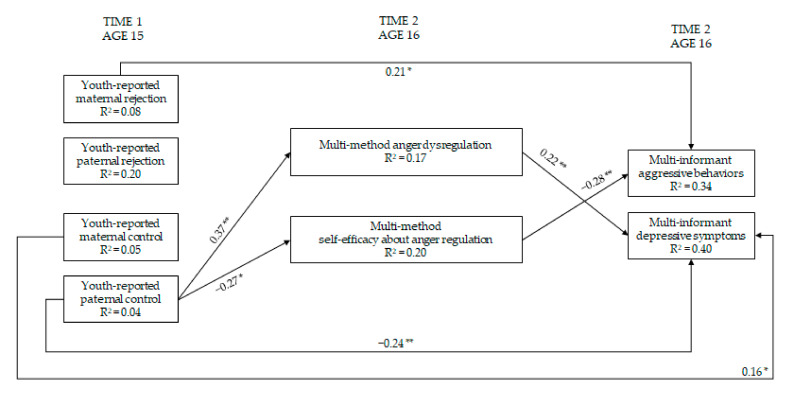
Models of relations of maternal and paternal rejection and control, regulation and self-efficacy regarding anger and youth adjustment (within-wave relations are not shown). Note: * = *p* ≤ 0.05; ** = *p* ≤ 0.01. Only significant paths are reported. Standardized coefficients are presented. For ease of interpretation, within-wave covariances and paths from parental education, youth sex, age, and social desirability are not depicted in the Figure 3 (see Supplementary Tables S1 and S2).

The first series of path analyses relied on the mediating role of anger-related measures in the association between maternal and paternal rejection and control, on the one side, and aggressive behaviors and depressive symptoms, on the other side, controlling for covariates (Figure 3). This model fitted the data well, χ^2^(11) = 12.19, *p* = 0.35, CFI = 0.99, RMSEA = 0.03, 90% CI = 0.00; 0.11, SRMR = 0.04, AIC = 2512.286. Stronger paternal control was associated with youth self-efficacy beliefs in dealing with anger, which was in turn associated with lower youth aggressive behaviors. In addition, stronger paternal control was associated with more youth anger dysregulation, which was in turn associated with more youth depressive symptoms. Moreover, stronger maternal rejection was directly associated with more youth aggressive behaviors. Stronger maternal control and lower paternal control were directly associated with more youth depressive symptoms. 

Indirect effects were also examined. The unstandardized indirect effect of paternal control on aggressive behaviors through youth self-efficacy in dealing with anger and the unstandardized indirect effect of paternal control on depressive symptoms through youth anger dysregulation were both significant (*b* = 0.02, SE = 0.11; 95% CI = 0.003; 0.05 and *b* = 0.03, SE = 0.02; 95% CI = 0.01; 0.08, respectively).

### 3.4. Maternal and Paternal Rejection and Control → Youth Adjustment → Regulation and Self-Efficacy Regarding Anger

**Figure 4:**
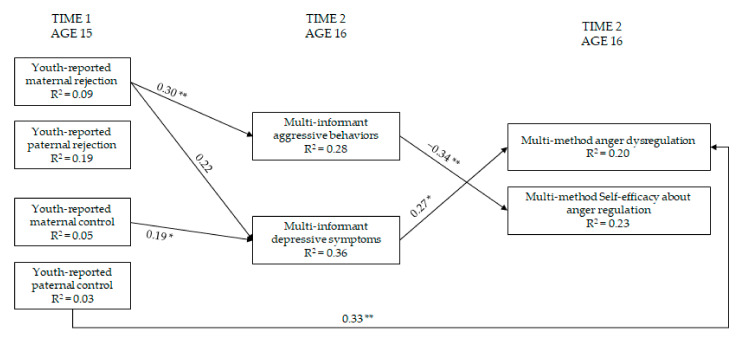
Models of relations of maternal and paternal rejection and control, youth adjustment, and regulation and self-efficacy regarding anger (within-wave relations are not shown). Note: * = *p* ≤ 0.05; ** = *p* ≤ 0.01. Only significant paths are reported. Standardized coefficients are presented. For ease of interpretation, within-wave covariances and paths from parental education, youth sex, age, and social desirability are not depicted in the Figure 4 (see Supplementary Tables S1 and S2).

A similar path analysis to the aforementioned one was then examined to consider the anger-related measures as outcomes (Figure 4). The model with youth adjustment as mediator fit the data, χ^2^(16) = 18.17, *p* = 0.31, CFI = 0.99, RMSEA = 0.04, 90% CI = 0.00; 0.10, SRMR = 0.05, AIC = 2508.267. This model describes the data better than the previous model, and it provided a lower (i.e., better) AIC. Stronger maternal rejection was associated with more youth aggressive behaviors, that in turn were significantly related to lower youth self-efficacy in dealing with anger. In addition, stronger maternal rejection was also significantly related to more youth depressive symptoms, that in turn were associated with more youth anger dysregulation. Stronger maternal control was associated with more depressive symptoms. Stronger paternal control was directly associated with more youth anger dysregulation. 

The unstandardized indirect effects of both maternal rejection and maternal control on youth anger dysregulation through youth depressive symptoms were not significant (*b* = 0.13, SE = 0.106; 95% CI = −0.004; −0.42 and *b* = 0.06, SE = 0.45; 95% CI = −0.003; −0.17, respectively). The unstandardized indirect effect of maternal rejection on youth self-efficacy regarding anger regulation through youth aggressive behaviors was significant (*b* = −0.25, SE = 0.127; 95% CI = −0.55; −0.05).

### 3.5. Maternal and Paternal Rejection and Control → Regulation and Self-Efficacy Regarding Sadness → Youth Adjustment

**Figure 5:**
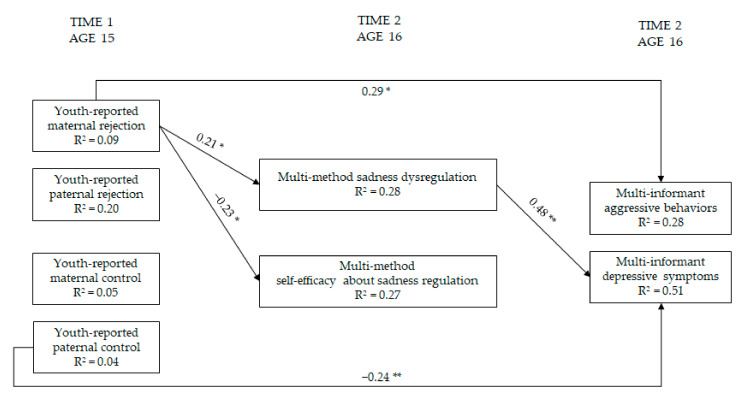
Model of relations of maternal and paternal rejection and control, regulation, and self-efficacy regarding sadness, and youth adjustment (within-wave relations are not shown). Note: * = *p* ≤ 0.05; ** = *p* ≤ 0.01. Only significant paths are reported. Standardized coefficients are presented. For ease of interpretation, within-wave covariances and paths from parental education, youth sex, age, and social desirability are not depicted in the Figure 5 (see Supplementary Tables S3 and S4).

The second series of path analyses focused on the role of sadness-related measures (Figure 5). These models were again examined while controlling for covariates. The model with sadness-related mediators fitted the data well, χ^2^(11) = 14.01, *p* = 0.23, CFI = 0.99, RMSEA = 0.05, 90%CI = 0.00; 0.12, SRMR = 0.05, AIC = 2442.717. Stronger maternal rejection was associated with lower youth self-efficacy beliefs regarding sadness regulation and more youth sadness dysregulation. The latter was in turn associated with more youth depressive symptoms. In addition, more maternal rejection was associated with more youth aggressive problems, whereas more paternal control was associated with fewer youth depressive symptoms. The unstandardized indirect effect of maternal rejection on depressive symptoms through youth sadness dysregulation was significant (*b* = 0.09, SE = 0.06; 95% CI = 0.001; 0.25).

### 3.6. Maternal and Paternal Rejection and Control → Youth Adjustment → Regulation and Self-Efficacy Regarding Sadness

**Figure 6:**
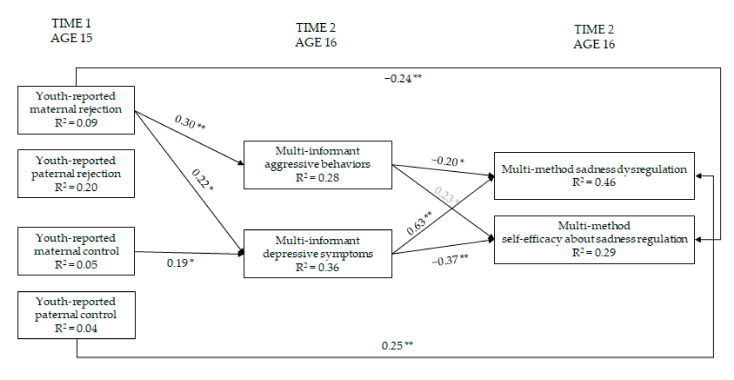
Model of relations of maternal and paternal rejection and control, youth adjustment, and regulation and self-efficacy regarding sadness (within-wave relations are not shown). Note: * = *p* ≤ 0.05; ** = *p* ≤ 0.01. Only significant paths are reported. Standardized coefficients are presented. For ease of interpretation, within-wave covariances and paths from parental education, youth sex, age, and social desirability are not depicted in the Figure 6 (see Supplementary Tables S3 and S4). The path coefficient reported in light grey shows a suppression effect. Indeed, in Table 2 the correlation between these variables is r = −0.11, *p* > 0.05.

In the model focused on youth adjustment as mediator in the association between parental rejection and control and sadness-related measures, controlling for the covariates, yielded a reasonable fit to the data, χ^2^(15) = 15.00, *p* = 0.45, CFI = 1.00, RMSEA = 0.00, 95% CI = 0.00; 0.09, SRMR= 0.05, AIC = 2435.710 (Figure 6). This sadness model describes the data better than the previous model, and it provided a lower (i.e., better) AIC. More maternal rejection was associated with more youth aggressive behaviors and depressive symptoms. In turn, more aggressive behaviors were associated with less youth sadness dysregulation and more youth self-efficacy beliefs regarding sadness regulation. In addition, more maternal rejection and control were related with higher youth depressive symptoms, that in turn were associated with more youth sadness dysregulation and less youth self-efficacy regarding sadness regulation. Finally, paternal control was directly related to more youth sadness dysregulation.

The unstandardized indirect effects of maternal rejection on youth sadness dysregulation through both youth aggressive behaviors and depressive symptoms were significant (*b* = −0.14, SE = 0.09; 95% CI = −0.41; −0.01 and *b* = 0.31, SE = 0.19; 95% CI = 0.02; 0.76, respectively). The unstandardized indirect effects of maternal rejection on youth self-efficacy regarding sadness regulation through both youth aggressive behaviors and depressive symptoms were significant (*b* = 0.15, SE = 0.11; 95% CI = 0.003; 0.47 and *b* = −0.12, SE = 0.09; 95% CI = −0.39; −0.004, respectively). Finally, the unstandardized indirect effects of maternal control on both youth sadness dysregulation and self-efficacy through youth depressive symptoms were significant (*b* = 0.14, SE = 0.08; 95% CI = 0.02; 0.34 and *b* = −0.05, SE = 0.04; 95% CI = −0.17; −0.004, respectively).

## 4. Discussion

The present study longitudinally investigated the association between parents’ rejection and control and adolescents’ aggressive problems and depressive symptoms, through specific mechanisms in youth emotion regulation, namely dysregulation and self-efficacy beliefs regarding anger and sadness. We focused on the perceptions that adolescents have of their mothers’ and fathers’ behaviors separately, while controlling for parental education, youth age, gender, and social desirability. Because emotion regulation related mechanisms and adjustment were measured concurrently, we compared two models: one model in which parenting behaviors were associated with dysregulation and self-efficacy, which in turn were associated with adjustment problems, and a second model in which parenting behaviors were related to adjustment problems, which in turn were related to dysregulation and self-efficacy. Both models were examined separately for anger and sadness.

Overall, the model that best represented the data was the one in which parenting behaviors were associated with adjustment problems, that in turn were associated with anger and sadness dysregulation, as well as with self-efficacy regarding anger and sadness regulation. In particular, focusing just on anger-related mechanisms, youth-reported maternal rejection at age 15 had a direct association with multi-informant (by mothers, fathers, and youths) youth aggressive behaviors at age 16. This result is consistent with other studies, including in Italy [28]. Adolescents’ aggressive problems in turn were associated with lower self-efficacy beliefs in regulating anger. This result is partly in agreement with studies highlighting an association between rejection, low warmth, and aggressive problems [28,29], and partly in agreement with studies suggesting that problematic outcomes are associated with a decrease in adolescents’ beliefs in being capable of dealing with anger [26]. Furthermore, this result is in line with Bandura (1997) [50] regarding the importance of mastery experiences in the development of self-efficacy. Thus, youths who engage more in aggressive behaviors presumably have lower mastery experiences related to their abilities to deal with anger, which in turn may have affected whether they believe themselves to be capable of handling their anger in stressful situations.

Although the model looking at dysregulation and self-efficacy regarding anger as outcomes was competitively better than the model that placed dysregulation and self-efficacy as mediators, the latter still had very good fit indices. In the latter model, of particular note is the role of paternal control as a risk factor for adolescent emotional development in relation to anger. In addition, whereas maternal control was associated with more depressive symptoms, paternal control was associated with less depressive symptoms one year later. Preliminary analysis rejected the possibility that this result was due to a suppression effect. Thus, in Italian culture in which mothers are known to be overly protective [51], mothers who are even more over-protective than the average Italian mother may lead their children to have a reduced sense of competence and a greater risk for internalizing symptoms [52,53]. In contrast, the amount of control exercised by Italian fathers was related to adolescents’ experience of more anger a year later (perhaps because adolescents felt they did not have enough space within which to develop a sense of autonomy), but at the same time, fathers’ control may have helped their children experience their fathers’ presence in their lives, contributing to fewer depressive symptoms one year later. This speculation needs further empirical evidence.

When focusing on sadness-related mechanisms, maternal rejection when adolescents were 15 years old was related to adolescents’ aggressive behaviors and depressive symptoms one year later. In turn, both adjustment problems were associated with higher sadness dysregulation and with lower self-efficacy beliefs regarding sadness regulation. In addition, maternal control was associated with higher later depressive symptoms, that in turn made those adolescents, on the one hand, feel more anger and, on the other hand, think themselves less capable of handling anger. As with anger related mechanisms, even though the model looking at dysregulation and self-efficacy regarding sadness as outcomes was competitively better than the model that placed dysregulation and self-efficacy as mediators, the latter still had very good fit indices. Specifically, in the latter, maternal rejection had a significant indirect effect on depressive symptoms through sadness dysregulation. This result is consistent with previous studies suggesting that children more rejected by their parents tend to develop more adjustment problems, especially depressive symptoms, perhaps because of reduced self-confidence, reduced problem-solving skills, and an increase in anxiety due to the paucity of positive interactions with their parents, and in particular with their mothers [54].

This study has several strengths. We employed many innovative efforts to clarify emotion-regulation processes and their predictors to advance researchers’ and practitioners’ abilities to prevent and act on the antecedents and outcomes of adolescents’ psychological maladjustment. We focused on anger and sadness because of the adverse consequences associated with their dysregulation, especially in adolescence [2,7]. In addition, we innovatively examined not only actual individuals’ behavior (i.e., anger and sadness regulation), but also their self-efficacy regarding anger and sadness regulation. This study is among the few exploring the joint contribution of both emotion regulation and related self-efficacy beliefs on adolescent adjustment problems. Relying on multiple-informants of youth aggressive problems and depressive symptoms is another strength of this study, in line with empirical evidence underlying the significant advantages of multiple-informant designs [45]. One of the biggest limitations of traditional methods to assess emotion regulation and self-efficacy regarding emotion regulation is that they lack ecological validity and information about the social context associated with emotional experience. To address this limitation, we focused on adolescent anger and sadness regulation and self-efficacy beliefs regarding anger and sadness, capitalizing on two methods: the traditional method of youth reports via questionnaire and the innovative method of mobile ecological momentary assessment, in which adolescents were prompted three times a day for 15 days via their cellphones to answer questions related to current emotions. The EMA method minimizes retrospective reporting biases and adds greater generalizability regarding emotional experience. Another strength was the examination of adolescent adjustment with a multi-informant approach.

This study is not without limitations that suggest relevant future research directions. First, emotion regulation related factors and adjustment problems were assessed concurrently; longer term longitudinal designs are needed to establish the bidirectional associations among parenting, emotion regulation related factors, and adolescent adjustment problems. Second, the use of self-reports may have introduced response biases and inflated the pattern of correlations. However, this limitation was minimized by two factors: (1) these results emerged after controlling for adolescents’ social desirability, (2) adjustment problems were not just youth-reported but reported by youth, mothers, and fathers. Future studies could combine adolescent and parent reports, also using observational methods. Third, the 100 families included in the present study cannot be considered representative of average Italian society, thus caution should be used in generalizing results from the present study to Italian populations, as well as to other populations from countries different from Italy, in particular concerning socio-economic stratification. Fourth, even though we relied upon adolescents’ daily reports about their emotion regulation-related indicators, we did not examine within-person daily associations in addition to between-person associations among emotions, regulation of specific emotions, and maladjustment. Future studies should include this level of analysis to establish how EMA analyses can build on the existing emotion, emotion regulation, and psychopathology literature. Fifth, the strong association between sadness dysregulation and depressive symptoms is in agreement with previous studies [11], but it may be also due to an overlapping item considered in both composite scores, namely “feeling sad and depressed”. Future studies may address this issue by considering other measures, beyond self-reports, to examine sadness and its association with depressive symptoms. Sixth, another problem of the analysis is the lack of control of the stability of the outcomes. Future studies should overcome such a limit and could also examine child effects on parenting. Thus, possible alternative models could be the use of T1 adolescent outcomes to predict T2 negative parenting.

## 5. Conclusions

In conclusion, those parents who, according to their adolescent children, rarely say something nice to them and do not pay attention to them, or that insist their children must do exactly as they are told, tend to have children who develop adjustment problems in adolescence, both in terms of aggressive behaviors and depressive symptoms. Those behaviors, in turn, are related to adolescents’ difficulties in dealing with anger and sadness, and to adolescents developing beliefs that they are not capable of dealing with such emotions. These findings could be useful for intervention programs focused on parent training to improve anger and sadness regulation skills and to decrease adolescent adjustment problems.

## Figures and Tables

**Table 1:** Correlations among the examined variables, means and standard deviations in the total sample.

		(1)	(2)	(3)	(4)	(5)	(6)	(7)	(8)	(9)	(10)	(11)	(12)	(13)	(14)
*Youth age: 15 years old*															
Maternal rejection via questionnaire	(1)	1													
Paternal rejection via questionnaire	(2)	0.48 **	1												
Maternal control via questionnaire	(3)	−0.09	−0.15	1											
Paternal control via questionnaire	(4)	−0.29 **	−0.14	0.31 **	1										
*Youth age: 16 years old*															
Youths’ anger dysregulation via questionnaire	(5)	0.18	0.09	0.07	0.21 *	1									
Youths’ anger dysregulation via mEMA	(6)	0.10	−0.09	0.09	0.21 *	0.16	1								
Youths’ sadness dysregulation via questionnaire	(7)	0.24 *	0.25 *	0.17	0.01	0.39 **	0.10	1							
Youths’ sadness dysregulation via mEMA	(8)	0.15	−0.02	0.09	0.14	0.21 *	0.89 **	0.26 **	1						
Youths’ self-efficacy about anger regulation via questionnaire	(9)	−0.15	−0.12	−0.06	−0.17	−0.21 *	−0.04	−0.21 *	−0.11	1					
Youths’ self-efficacy about anger regulation via mEMA	(10)	−0.13	−0.10	−0.10	−0.14	−0.19	−0.27 *	−0.28 **	−0.29 **	0.33 **	1				
Youths’ self-efficacy about sadness regulation via questionnaire	(11)	−0.21 *	−0.20	−0.03	0.13	0.06	−0.07	−0.45 **	−0.16	0.22 *	0.25 *	1			
Youths’ self-efficacy about sadness regulation via mEMA	(12)	−0.27 **	−0.19	0.02	0.15	−0.19	−0.26 *	−0.28 **	−0.28 **	0.09	0.52 **	0.19	1		
Youths’ aggressive behaviors	(13)	0.39 **	0.35 **	0.09	0.02	0.29 **	0.03	0.28 **	0.05	−0.35 **	−0.24 *	−0.04	−0.08	1	
Youths’ depressive symptoms	(14)	0.34 **	0.30 **	0.16	−0.17	0.25 *	0.20 *	0.67 **	0.26 **	−0.21 *	−0.20	−0.36 **	−0.12	0.56 **	1
Means		1.36	1.47	2.87	2.73	3.4	1.27	2.38	1.33	2.87	3.24	3.28	3.21	0.36	0.29
Standard deviation		0.36	0.43	0.67	0.76	0.64	0.41	0.98	0.46	0.81	0.81	0.85	0.86	0.19	0.31

Note: * = *p* ≤ 0.05; ** = *p* ≤ 0.01.

**Table 2:** Correlations among the examined variables including multi-method variables, means and standard deviations in the total sample.

		(1)	(2)	(3)	(4)
*Youth age: 15 years old*					
Maternal rejection via questionnaire		0.09	0.23 *	−0.19	−0.34 **
Paternal rejection via questionnaire		−0.03	0.13	−0.15	−0.26 *
Maternal control via questionnaire		0.08	0.16	−0.11	−0.03
Paternal control via questionnaire		0.30 **	0.10	−0.18	0.18
*Youth age: 16 years old*					
Z-score youths’ anger dysregulation multi-method	(1)	1			
Z-score youths’ sadness dysregulation multi-method	(2)	0.63 **	1		
Z-score youths’ self-efficacy about anger regulation multi-method	(3)	−0.21 *	−0.32 **	1	
Z-score youths’ self-efficacy about sadness regulation multi-method	(4)	−0.09	−0.44 **	0.43 **	1
Youths’ aggressive behaviors		0.16	0.20 *	−0.37 **	−0.11
Youths’ depressive symptoms		0.23 *	0.57 **	−0.25 **	−0.34 **
Means		−0.01	0.00	−0.01	−0.01
Standard deviation		0.80	0.79	0.84	0.80

Note: * = *p* ≤ 0.05; ** = *p* ≤ 0.01. Multi-Method refers to those scores created by averaging the standardized correspondent scores via questionnaire and via mEMA.

## Data Availability

Data available on request due to restrictions, e.g., privacy or ethical. The data presented in this study are available on request from the corresponding author. The data are not publicly available due to the privacy and professional specificity of the people who took part in this research.

## Supplementary Materials

The following are available online at https://www.mdpi.com/article/10.3390/ijerph19042251/s1, Table S1: Within-time relations from the model of maternal and paternal rejection and control, regulation and self-efficacy about anger, and youth adjustment. Table S2: Standardized coefficients of the associations of parental education, youth gender, age, and social desirability with the examined variables from the model of maternal and paternal rejection and control, regulation and self-efficacy about anger, and youth adjustment. Table S3: Within-time relations from the model of maternal and paternal rejection and control, regulation and self-efficacy about sadness, and youth adjustment. Table S4: Standardized coefficients of the associations of parental education, youth gender, age, and social desirability with the examined variables from the model of maternal and paternal rejection and control, regulation and self-efficacy about sadness, and youth adjustment.
