# Supplementary material for: Negative Parenting, Adolescents’ Emotion Regulation, Self-Efficacy in Emotion Regulation, and Psychological Adjustment
Source: Int J Environ Res Public Health. 2022 Feb 16;19(4):2251. doi: 10.3390/ijerph19042251 (PMC8871997; doi:10.3390/ijerph19042251)
Supplement: Supplementary file 1 [file ijerph-19-02251-s001.zip › ijerph-1547008-supplementary.pdf]

Supplementary

Table S1. Within-time relations from the model of maternal and paternal rejection and control, regulation and self-efficacy about anger, and youth adjustment.

	Estimate	*SE*
Within-Time 1 relations		
Youth's reported maternal rejection ↔ Youth's reported paternal rejection	0.46 **	0.08
Youth's reported maternal rejection ↔ Youth's reported maternal control	−0.12	0.09
Youth's reported maternal rejection ↔ Youth's reported paternal control	−0.25 **	0.09
Youth's reported paternal rejection ↔ Youth's reported maternal control	−0.16	0.10
Youth's reported paternal rejection ↔ Youth's reported paternal control	−0.07	−0.10
Youth's reported maternal control ↔ Youth's reported paternal control	0.29 **	0.08
Within-Time 2 relations		
Multi-method anger dysregulation ↔ Multi-method self-efficacy about anger regulation	−0.53	0.10
Multi-informant aggressive behaviors ↔ Multi-informant depressive symptoms	0.44 **	0.08

Note: Standardized coefficients are reported. * = $p \leq 0.05$; ** = $p \leq 0.01$.

Table S2. Standardized coefficients of the associations of parental education, youth gender, age, and social desirability with the examined variables from the model of maternal and paternal rejection and control, regulation and self-efficacy about anger, and youth adjustment.

	**Estimate**	***SE***
Maternal education →		
Youth's reported maternal rejection Time 1	0.18	0.14
Youth's reported paternal rejection Time 1	0.08	0.14
Youth's reported maternal control Time 1	−0.90	0.14
Youth's reported paternal control Time 1	−0.93	0.15
Multi-method self-efficacy about anger regulation Time 2	−0.15	0.12
Multi-method anger dysregulation Time 2	0.27 *	0.12
Multi-informant aggressive behaviours Time 2	−0.15	0.12
Multi-informant depressive symptoms Time 2	−0.06	0.14
Paternal education →		
Youth's reported maternal rejection Time 1	−0.16	0.15
Youth's reported paternal rejection Time 1	−0.07	0.16
Youth's reported maternal control Time 1	−0.10	0.16
Youth's reported paternal control Time 1	0.01	0.17
Multi-method self-efficacy about anger regulation Time 2	0.29 *	0.13
Multi-method anger dysregulation Time 2	−0.18	0.14
Multi-informant aggressive behaviours Time 2	0.10	0.13
Multi-informant depressive symptoms Time 2	−0.11	0.18
Youth gender →		
Youth's reported maternal rejection Time 1	0.07	0.10
Youth's reported paternal rejection Time 1	0.27 **	0.09
Youth's reported maternal control Time 1	−0.05	0.10
Youth's reported paternal control Time 1	−0.10	0.10
Multi-method elf-efficacy about anger regulation Time 2	−0.09	0.10
Multi-method anger dysregulation Time 2	0.03	0.10
Multi-informant aggressive behaviours Time 2	0.09	0.09
Multi-informant depressive symptoms Time 2	0.34 **	0.09
Youth age →		
Youth's reported maternal rejection Time 1	0.07	0.10
Youth's reported paternal rejection Time 1	0.27 **	0.09
Youth's reported maternal control Time 1	0.02	0.10
Youth's reported paternal control Time 1	−0.12	0.09
Multi-method self-efficacy about anger regulation Time 2	0.16	0.08
Multi-method anger dysregulation Time 2	−0.06	0.09
Multi-informant aggressive behaviours Time 2	0.12	0.09
Multi-informant depressive symptoms Time 2	0.09	0.84
Youth social desirability →		
Youth's reported maternal rejection Time 1	−0.24 *	0.09
Youth's reported paternal rejection Time 1	−0.30 **	0.09
Youth's reported maternal control Time 1	−0.12	0.10
Youth's reported paternal control Time 1	0.01	0.10
Multi-method self-efficacy about anger regulation Time 2	0.14	0.10
Multi-method anger dysregulation Time 2	0.00	0.10
Multi-informant aggressive behaviours Time 2	−0.18	0.09
Multi-informant depressive symptoms Time 2	−0.19 *	0.09

Note: * = $p \leq 0.05$; ** = $p \leq 0.01$.

Table S3. Within-time relations from the model of maternal and paternal rejection and control, regulation and self-efficacy about sadness, and youth adjustment.

	Estimate	*SE*
Within-Time 1 relations		
Youth's reported maternal rejection ↔ Youth's reported paternal rejection	0.46 **	0.08
Youth's reported maternal rejection ↔ Youth's reported maternal control	−0.13	0.10
Youth's reported maternal rejection ↔ Youth's reported paternal control	−0.26 **	0.10
Youth's reported paternal rejection ↔ Youth's reported maternal control	−0.17	0.10
Youth's reported paternal rejection ↔ Youth's reported paternal control	−0.07	0.10
Youth's reported maternal control ↔ Youth's reported paternal control	0.28 **	0.10
Within-Time 2 relations		
Multi-method sadness dysregulation ↔ Multi-method self-efficacy about sadness regulation	−0.30 **	0.09
Multi-informant aggressive behaviours ↔ Multi-informant depressive symptoms	0.44 **	0.08

Note: Standardized coefficients are reported. * = $p \leq 0.05$; ** = $p \leq 0.01$.

Table S4. Standardized coefficients of the associations of parental education, youth gender, age, and social desirability with the examined variables from the model of maternal and paternal rejection and control, regulation and self-efficacy about sadness, and youth adjustment.

	Estimate	*SE*
Maternal education →		
Youth's reported maternal rejection Time 1	0.20	0.13
Youth's reported paternal rejection Time 1	0.01	0.14
Youth's reported maternal control Time 1	−0.09	0.14
Youth's reported paternal control Time 1	−0.12	0.15
Multi-method self-efficacy about sadness regulation Time 2	0.02	0.12
Multi-method sadness dysregulation Time 2	0.31 **	0.10
Multi-informant aggressive behaviours Time 2	−0.17	0.12
Multi-informant depressive symptoms Time 2	−0.08	0.14
Paternal education →		
Youth's reported maternal rejection Time 1	−0.17	0.15
Youth's reported paternal rejection Time 1	−0.08	0.16
Youth's reported maternal control Time 1	−0.11	0.16
Youth's reported paternal control Time 1	0.05	0.17
Multi-method self-efficacy about sadness regulation Time 2	−0.03	0.13
Multi-method sadness dysregulation Time 2	−0.19 **	0.12
Multi-informant aggressive behaviours Time 2	0.13	0.13
Multi-informant depressive symptoms Time 2	−0.08	0.18
Youth gender →		
Youth's reported maternal rejection Time 1	0.07	0.10
Youth's reported paternal rejection Time 1	0.27 **	0.09
Youth's reported maternal control Time 1	−0.05	0.10
Youth's reported paternal control Time 1	−0.11	0.10
Multi-method self-efficacy about sadness regulation Time 2	−0.17 *	0.09
Multi-method sadness dysregulation Time 2	0.16 *	0.08
Multi-informant aggressive behaviours Time 2	0.08	0.09
Multi-informant depressive symptoms Time 2	0.34 **	0.09
Youth age →		
Youth's reported maternal rejection Time 1	0.02	0.09
Youth's reported paternal rejection Time 1	0.15	0.09
Youth's reported maternal control Time 1	0.02	0.10
Youth's reported paternal control Time 1	−0.13	0.10
Multi-method self-efficacy about sadness regulation Time 2	0.14	0.08
Multi-method sadness dysregulation Time 2	0.03	0.08
Multi-informant aggressive behaviours Time 2	0.13	0.09
Multi-informant depressive symptoms Time 2	0.09	0.08
Youth social desirability →		
Youth's reported maternal rejection Time 1	−0.24 *	0.09
Youth's reported paternal rejection Time 1	−0.30 **	0.09
Youth's reported maternal control Time 1	−0.12	0.09
Youth's reported paternal control Time 1	0.00	0.10
Multi-method self-efficacy about sadness regulation Time 2	0.29 **	0.09
Multi-method sadness dysregulation Time 2	−0.09	0.08
Multi-informant aggressive behaviours Time 2	−0.17 *	0.09
Multi-informant depressive symptoms Time 2	−0.19 *	0.09

Note: * = $p \leq 0.05$; ** = $p \leq 0.01$.
